# Subclinical Changes in Type 2 Diabetes Patients with Heart Failure Stage A and B Treated with Oral Semaglutide

**DOI:** 10.3390/medicina61040567

**Published:** 2025-03-22

**Authors:** Larissa Dăniluc, Adina Braha, Oana Elena Sandu, Carina Bogdan, Loredana Suhov, Lina Haj Ali, Alexandra-Iulia Lazăr-Höcher, Alexandra Sima, Adrian Apostol, Mihaela Viviana Ivan

**Affiliations:** 1 Doctoral School, “Victor Babes” University of Medicine and Pharmacy, Eftimie Murgu Sq. No. 2, 300041 Timisoara, Romania; larissa.daniluc@umft.ro (L.D.); oana.ciolpan@umft.ro (O.E.S.); carina.bogdan@umft.ro (C.B.); loredana.ogarcin@umft.ro (L.S.); lina.haj-ali@umft.ro (L.H.A.); alexandra.hocher@umft.ro (A.-I.L.-H.); 2 Department of Cardiology, Pius Brinzeu Clinical Emergency County Hospital Timisoara, 300736 Timisoara, Romania; adrian.apostol@umft.ro (A.A.); ivan.viviana@umft.ro (M.V.I.); 3 Department VII, Internal Medicine II, Discipline of Cardiology, “Victor Babes” University of Medicine and Pharmacy, Eftimie Murgu Sq. No. 2, 300041 Timişoara, Romania; 4 Department of Second Internal Medicine Diabetes, Nutrition, Metabolic Diseases, and Systemic Rheumatology, “Victor Babes” University of Medicine and Pharmacy, 300041 Timisoara, Romania; sima.alexandra@umft.ro; 5 Department of Diabetes, Nutrition and Metabolic Diseases Clinic, “Pius Brînzeu” Emergency Clinical County University Hospital, 300723 Timisoara, Romania; 6 Centre for Molecular Research in Nephrology and Vascular Disease/MOL-NEPHRO-VASC, “Victor Babes” University of Medicine and Pharmacy, 300041 Timisoara, Romania; 7 Institute of Cardiovascular Diseases Timisoara, 300310 Timisoara, Romania

**Keywords:** speckle-tracking echocardiography, GLP-1 receptor agonists, visceral adiposity, metabolic control

## Abstract

*Background and Objectives*: Heart failure (HF) among patients with type 2 diabetes (T2DM) is linked to significant morbidity and mortality, despite the increased availability of new drug therapy. This study aims to investigate subclinical changes in patients with HF stage A (at risk for HF) and B (Pre-HF) and T2DM treated with oral semaglutide. *Materials and Methods*: In a prospective, observational, single-center study, 50 T2DM patients were assessed at baseline and one-year follow-up for changes in spectral Doppler, tissue Doppler, and speckle-tracking (2DST) and metabolic parameters. *Results*: Correlation and regression analyses identified predictors of Δ GLS. In correlation analysis, Δ GLS showed a negative correlation with Δ VAI (rho = −0.3, *p* = 0.02), Δ LAP (rho = −0.3, *p* = 0.04), Δ FPG (rho = −0.3, *p* = 0.009), Δ TG (rho = −0.4, *p* = 0.004), and Δ TyG (rho = −0.3, *p* = 0.02). In linear stepwise regression analysis, the most accurate model, with a *p*-value < 0.001, was M_3_, explaining 70% of the variance in Δ GLS (adjusted R^2^ = 0.7); this model included Δ FPG (beta −0.4, *p* = 0.001), Δ CRR (beta −1.3, *p* < 0.001), and Δ LDLc (beta 0.6, *p* = 0.01). *Conclusions:* These findings show that improved subclinical left ventricular systolic dysfunction is associated with improved glycemic control, visceral adiposity, and reduced insulin resistance, respectively, with improved lipid profiling.

## 1. Introduction

Type 2 diabetes mellitus (T2DM) is an important metabolic disorder impacting more than 536 million people globally (reported by the International Diabetes Federation) [1,2]. The global prevalence of T2DM has been rising consistently, and its correlation with cardiovascular diseases (CVDs) is well documented [3]. The prevalence of heart failure (HF) in patients with T2DM is estimated at 9–22%, which is four times greater than in the non-diabetic population [4,5].

Hyperglycemia starts a cascade of molecular alterations that cause myocardial fibrosis, increased stiffness, and cardiac contractility dysfunction, finally leading to heart failure if not managed effectively [2]. HF is still linked to significant morbidity and mortality, despite the increased availability of new drug therapy [6]. In order to prevent disease development, enable prompt and suitable therapeutic management, and decrease mortality rates, it is essential to enhance comprehension of the interaction between T2DM and the cardiovascular system. Recognizing early indicators of cardiac dysfunction in T2DM is crucial for implementing preventive strategies and optimizing patient outcomes [2]. HF has been classified into four stages: A, B, C, and D, by the American College of Cardiology (ACC) and the American Heart Association (AHA). In summary, the stages reflect a progression from being at risk (Stage A), to having structural heart disease without symptoms, known as pre-heart failure (Stage B), to symptomatic heart failure (Stage C), and ultimately, to advanced, refractory heart failure (Stage D) [7]. Stage A patients are those with biological and behavioral cardiovascular risk factors, such as T2DM, obesity, and hypertension, but without established structural heart disease and without symptoms. Pre-heart failure (PHF) is defined by identifying a structural or functional heart disease or abnormally elevated cardiac biomarkers in patients without cardiovascular symptoms [8].

Among the mentioned risk factors, obesity plays an important role, frequently coexisting with T2DM and increasing cardiovascular risk. Obesity contributes substantially to the global mortality associated with CVD [9]. In obese patients, various pathophysiological processes, like neurohormonal activation, hemodynamic alterations, hormonal effects of dysfunctional adipose tissue, and microvascular dysfunction, can lead directly to an increased PHF risk. Being overweight and obese may indirectly cause PHF through the development of hypertension, dyslipidemia, metabolic syndrome, and T2DM by causing insulin resistance [6]. Echocardiographic changes, such as left ventricular (LV) hypertrophy, left atrial enlargement, cardiac fibrosis, and diastolic dysfunction, which may progress to overt HF, have been identified in previous studies in patients with obesity [10,11]. Further exploration is required regarding the subclinical impairment of left ventricular systolic dysfunction in obese patients.

Targeting HF prior to its clinical manifestation, specifically at the stage of PHF, during the asymptomatic phase, is an important area of research. The subclinical phase presents an opportunity for early intervention to prevent the progression to more severe HF [12]. Knowledge gaps exist regarding the appropriate timing for initiating drug therapy, despite the enhanced availability of the four pillars of pharmacotherapy for HF treatment [6,13]. The subclinical phase offers an opportunity for early intervention to prevent further progression to more severe HF. Echocardiography is a valuable tool for diagnosing, monitoring, and managing CVD, due to its ease of use, low radiation exposure, and portability. Echocardiographic techniques may provide a significant non-invasive approach to identify subclinical LV systolic dysfunction, including Tissue Doppler Imaging (TDI) and global longitudinal strain (GLS), evaluated by 2D speckle-tracking echocardiography (2D-STE). They are considered more sensitive in evaluating systolic function than ejection fraction, and are also valuable for assessing diastolic function, offering a comprehensive evaluation of myocardial performance. In the stepwise approach to patients with a predisposition for HF, 2D STE plays an essential role in those patients in stage A and stage B for early identification of an underlying cardiomyopathy [14]. GLS provides independent prognostic information regarding the long-term risk of cardiovascular morbidity and mortality [15].

The present study aims to investigate the dynamics of these subclinical LV systolic and diastolic dysfunction in patients with T2DM treated for one year with oral semaglutide, in association with changes in anthropometric indexes, insulin resistance markers, atherogenic indexes, and metabolic biomarkers.

## 2. Materials and Methods

### 2.1. Study Population

We conducted a prospective study, recruiting patients from October 2022 and evaluating them at baseline and after one year of treatment with oral semaglutide. Oral semaglutide was an add-on therapy to the standard of care provided by their diabetologist. Patients were recruited from the Diabetes Center, and evaluated both in the Diabetes Center and in the Cardiology Clinic of the University Emergency County Hospital Pius Brînzeu Timisoara, Romania.

### 2.2. Inclusion and Exclusion Criteria

Patients were selected based on the following inclusion criteria: T2DM patients with an age over 18 years, left ventricular ejection fraction (LVEF) > 40%, and an estimated glomerular filtration rate (eGFR) > 15 mL/min/1.73 m^2^. The exclusion criteria were atrial and ventricular arrhythmias, acute and chronic coronary syndrome, significant cardiovascular valvulopathies, previously known HF or a history of ischemic stroke, ongoing or planned pregnancy, lactation, a type other than T2DM, underweight with a body mass index (BMI) of <25 kg/m^2^, or refusal to participate in this clinical trial. After applying the inclusion and exclusion criteria, 72 patients were enrolled in the study. However, during the study, 22 patients were lost to follow-up (missing at medical assessments) or did not tolerate oral semaglutide until the end of the study, and were excluded from the final analysis. The intolerance to oral semaglutide was primarily due to gastrointestinal side effects, which are common with GLP-1 receptor agonists. These side effects included nausea, vomiting, diarrhea, and abdominal pain.

### 2.3. Laboratory Tests and Clinical Examination

For a complete evaluation of cardiovascular risk, anthropometric data are integrated with laboratory test results. Anthropometric measurements included BMI and waist circumference, measured at the midpoint between the lowest rib and the top of the iliac crest. Furthermore, routine blood tests (following a minimum of 12 h of food abstinence), such as glycated hemoglobin A1c (HbA1c), lipid panels (triglycerides-TG, high-density lipoprotein cholesterol-HDLc, low-density lipoprotein cholesterol-LDLc, total cholesterol (TC)), and markers of kidney function, such as eGFR and urinary albumin/creatinine ratio (UACr), provided essential data on metabolic control and organ function.

Used definitions:

LAP (lipid accumulation product) = (WC − 58) × TG for women and (WC − 65) × TG for men [16]. WC values below 65/58 cm in men/women were reassigned for 66.0/59.0 cm to avoid invalid data [17]. VAI, LAP, TG, and HDL-C are presented as mmol/L [18].

Body mass index (BMI) = weight/height^2^

Body surface area = Weight^0.425^ × Height^0.725^ × 0.007184 [19]

Conicity index = 0.109^−1^ × waist (meters) × (weight [kg]/height [meters]) − 1/2 [20]

Atherogenic index of plasma (AIP) = log [TG/HDLc] [21]

Cardiac risk ratio (CRR) = TC/HDLc [22]

Atherogenic coefficient (AC) = non-HDLc/HDLc [21]

Product of triglycerides and glucose (TyG) = ln [Fasting triglyceride (mg/dL) × Fasting glucose (mg/dL)]/2 [23]

Visceral adiposity index (VAI): [24]

Females’ VAI = (waist/(36.58 + (1.89 × BMI))) × (TG/0.81) × (1.52/HDLc), males’ VAI = (waist/(39.68 + (1.88 × BMI))) × (TG/1.03) × (1.31/HDLc), where the waist is expressed in cm, BMI in kg/m^2^, and TG and HDLc in mmol/L.

### 2.4. Cardiology Assessment

Each participant underwent a complete cardiology evaluation during the study, including a detailed clinical history and a physical examination. Blood pressure was measured under optimal conditions, according to current guidelines, to detect hypertension, with the patient sitting and resting for at least 5 min. An electrocardiogram (ECG) was performed to assess cardiac rhythm and identify potential abnormalities. Two cardiologists performed transthoracic echocardiography using Esaote S.p.A. MyLabX8 eXP (Via Enrico Melen 77, 16152 Genova, Italy, 2021) to evaluate cardiac structure and function.

Echocardiographic measurements included left ventricular dimensions, such as interventricular septum diameter (IVS), posterior wall thickness (PWT), left ventricular end-diastolic diameter (LVEDD), and left atrial diameter (LA), obtained in the parasternal long-axis view to assess cardiac remodeling and hypertrophy. Left atrial volume (LAv) was determined using Simpson’s biplane method from the apical four-chamber view. It was indexed to body surface area (BSA) to account for individual variability, providing a robust marker of diastolic dysfunction and elevated filling pressures. Epicardial fat tissue (EAT), considered a substitute indicator of visceral adiposity, was assessed posteriorly in the parasternal long-axis view. Diastolic function was comprehensively evaluated by pulsed-wave Doppler analysis of mitral inflow velocities (E and A waves) and their ratio (E/A), alongside TDI of mitral annular velocities (e′ and a′), enabling calculation of the E/e′ ratio, a key marker of LV filling pressures. Left ventricular end-diastolic volume (LVEDD) and left ventricular ejection fraction (LVEF) were calculated using Simpson’s method in the apical four-chamber view. The systolic function and stroke volume are correlated with the mitral annulus’s degree of systolic mobility. The mitral annulus’s systolic velocity (S′) is often greater than 6 cm/s. GLS was measured using speckle-tracking echocardiography from apical views (2-, 3-, and 4-chamber views), offering a sensitive index for subclinical LV systolic dysfunction. The endocardial border was automatically traced for the region of interest at the end-systole, and subsequently reviewed and adjusted manually to ensure precise speckle-tracking. If speckle-tracking was not feasible from a specific view, the GLS was determined by averaging measurements from the other accessible views. The American Society of Echocardiography (ASE)/European Association of Cardiovascular Imaging (EACVI) Task Force established absolute values for GLS ranging from −18.0% to −21.5% [25]. M-mode echocardiography assessed right ventricular function by measuring tricuspid annular plane systolic excursion (TAPSE). Pulmonary artery acceleration time (PAAT) and peak velocity (PV) were evaluated in the parasternal short-axis view to screen for pulmonary hypertension and to identify increased right ventricular afterload.

### 2.5. Ethical Consideration

The hospital ethics committee approved the study (43/26 September 2022), confirming its adherence to the Helsinki Declaration (2013) and General Data Protection Regulation (GDPR) compliance regarding patient data confidentiality. All participants provided written informed consent before enrollment.

### 2.6. Statistical Analysis

The statistical analysis was performed with JASP version 0.19.0 (University of Amsterdam, The Netherlands; https://jasp-stats.org; 8 January 2025). All variables of interest were input in basic descriptive analysis. The distribution of the data points was tested using the Shapiro–Wilk test. The continuous variables with Gaussian distribution were described as means and standard deviations. In contrast, the central tendency of the non-parametric variables was presented as the median, with the dispersion as the minimum and maximum values of the data points. In order to compare independent samples by a factor, Student’s *t*-test and the Mann–Whitney U test were applied, depending on the distribution of the analyzed variables. The changes in variables during the study assessments are presented as Δ between the first and last assessments. The associations between the changes in variables during the study were evaluated in a correlation analysis, and the correlation coefficients Pearson’s r and Spearman rho were presented and displayed in a matrix of plots between continuous variables with significant associations. We performed linear regression analysis backward and stepwise to model a linear relationship between one or more explanatory predictors and a continuous dependent variable. The modeling results were presented with the adjusted R-squared value, root mean square error (RMSE), *p*-value for each covariate predictor, and *p*-value, model coefficients, and t-value for the model (ANOVA). The significance threshold was set as *p* < 0.05.

## 3. Results

The final analysis included 50 patients, of whom 46% (23/50) were male. The patients had a mean age of 58.8 ± 10.4 years and a diabetes duration of a median of 7 years (0;21), with no differences across genders. All patients were overweight, with different degrees of obesity, at the study enrollment. Men had a higher weight, abdominal waist, and conicity index than women. However, the BMI was similar regardless of gender, with an overall mean of 34.3 ± 6.4 kg/m^2^. All patients were on a moderate statin dose and had a mean LDLc of 107.6 ± 38.0 mg/dL, with marginal differences between men and women (Table 1).

At the follow-up, patients who remained on treatment with oral semaglutide at the maximum tolerated dose presented a mean decrease in weight of −5.6 kg, and a mean decrease in abdominal waist of −4.7 cm, resulting in a significantly lower BMI by −2 kg/m^2^, and a lower conicity index and LAP (Table 2). Although LDLc and HDLc did not reach statistical significance, overall, the TC was significantly reduced, with a median of 21.9 mg/dL. The dynamics of lipid particles also influenced the CRR and AC, resulting in improved atherogenic indexes. Moreover, the glycemic control was significantly improved, reflected by lower FPG, with a mean of −27.7 mg/dL, and lower HbA1c, with a mean of −1.1%. The improved metabolic control was also reflected by a reduction in the mean TyG from 5.0 ± 0.3 to 4.9 ± 0.2 (*p* = 0.001, Table 2).

The dynamics of the cardiac parameters studied showed a significant improvement in EAT, by −0.3 mm (*p* = 0.0005), and in MV e′ septum, with a mean of −1 (*p* = 0.03). Also, the GLS was significantly higher after one year of treatment with oral semaglutide, increasing from −16.1 ± 1.6% to −16.8 ± 1.9% (*p* < 0.0001, Table 2).

**Figure 1:**
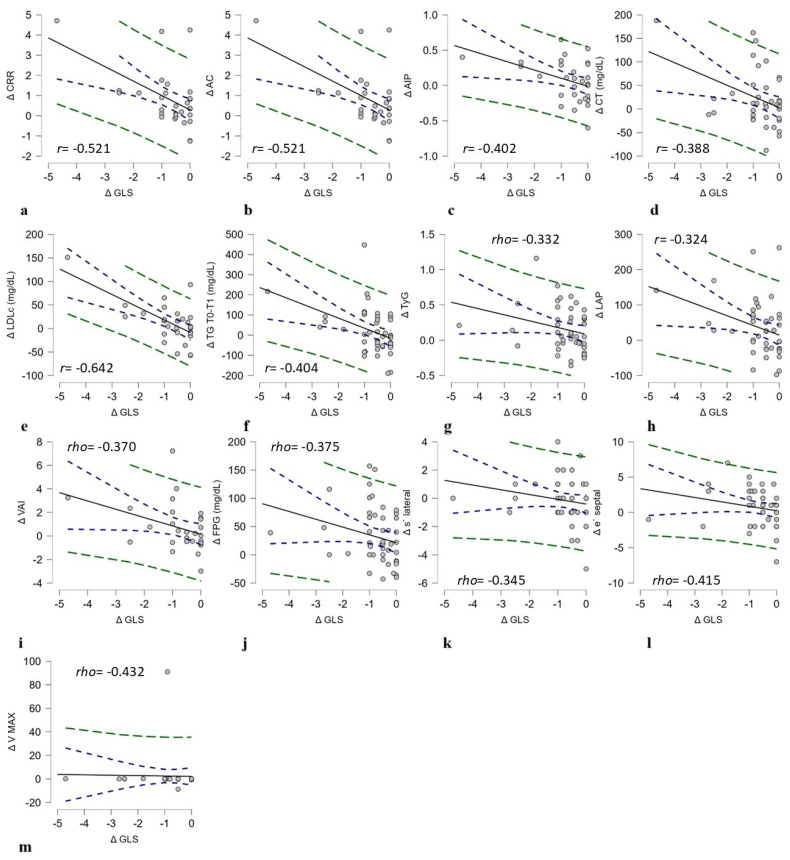
A graphical representation of the significant correlations between changes in variables and Δ GLS in patients with type 2 diabetes treated for one year with oral semaglutide. Subfigures (**a**–**m**) shows a negative correlation between Δ GLS and Δ CRR (**a**), Δ AC (**b**), Δ AIP (**c**), Δ CT (**d**), Δ LDLc (**e**), Δ TG (**f**), Δ TyG (**g**), Δ LAP (**h**), Δ VAI (**i**), Δ FPG (**j**), Δ s′ lateral wall (**k**), Δ e′ septal wall (**l**), and Δ PV PA (**m**). The green dashed line represents the 95% prediction intervals, and the blue dashed lines represent the 95% confidence intervals. Each plot presents the correlation coefficient Pearson r (if the association was linear) or Spearman rho (if the association was monotonic) to quantify the association between variables.

We performed a correlation analysis to evaluate the association between the changes in all parameters that reached statistical significance. Δ GLS showed a negative correlation with Δ VAI (rho = −0.3, *p* = 0.02), Δ LAP (rho = −0.3, *p* = 0.04), Δ FPG (rho = −0.3, *p* = 0.009), Δ TG (rho = −0.4, *p* = 0.004), and Δ LDLc (r = −0.6, *p* < 0.001). The negative correlation between Δ TyG and Δ GLS (rho = −0.3, *p* = 0.02) suggests that improved insulin sensitivity (lower TyG) is associated with better GLS. The AC, which reflects the balance between pro-atherogenic and anti-atherogenic cholesterol fractions, had a moderate negative correlation with Δ GLS. These results show that the improvement of subclinical left ventricular systolic dysfunction is associated with improved glycemic control and a decrease in triglyceride levels and LDLc, indicating improved visceral adiposity and reduced insulin resistance (Table 3, Figure 1).

In terms of speckle-tracking echocardiography parameters, Δ GLS did not show an association with Δ EAT (mm) or Δ E/A, but correlated indirectly with Δ s′ lateral wall (rho = −0.3, *p* = 0.02), Δ e′ septal wall (rho = −0.4, *p* = 0.005), and Δ PV PA (rho = −0.4, *p* = 0.01), as presented in Table 4, Figure 1.

Table 5 presents the results of a backward-manner linear regression analysis aimed at identifying the predictive factors of changes in GLS in studied patients. In this regression model analysis, we included all potential predictive parameters and used a backward manner that removed the least significant parameter until only the significant predictors remained. Model 0 is the null model before any parameters are removed and serves as a baseline model. The most accurate model, with a *p*-value of 0.008, was M_3_, explaining 90% of the variance in Δ GLS (adjusted R^2^ = 0.9), with the lowest RMSE of 0.3. The M_3_ model included Δ FPG (beta −0.7, *p* = 0.01), Δ BMI (beta −2.5, *p* = 0.04), Δ Weight (beta 1.9, *p* = 0.06), Δ Conicity index (beta −1.1, *p* = 0.01), Δ CRR (beta −2.3, *p*< 0.001), Δ Waist (beta 1.1, *p* = 0.009), Δ LAP (beta −1.7, *p* = 0.009), Δ EAT (beta 0.4, *p* = 0.05), Δ HbA1c (beta 0.4, *p* = 0.01), Δ HDLc (beta −1.2, *p* = 0.007), Δ LDLc (beta 1, *p* = 0.01), Δ LVEF (beta −0.4, *p* = 0.06), Δ PWT (beta 0.7, *p* = 0.02), Δ s′ septal (beta 0.6, *p* = 0.03), Δ s′ lateral (beta −0.7, *p* = 0.01), Δ IVS (beta −0.8, *p* = 0.02), and Δ TG (beta 2.6, *p* = 0.006).

We repeated the linear regression analysis stepwise, adding a predictor based on a *p*-value of 0.05 and removing it if the *p*-value was 0.1. Table 6 presents the results of this stepwise linear regression analysis, which aimed to identify the true predictive factors of changes in GLS in the studied patients. The most accurate model, with a *p*-value < 0.001, was M_3_, explaining 70% of the variance in Δ GLS (Adjusted R^2^ = 0.7), with the lowest RMSE of 0.5. The M_3_ model included Δ FPG (beta −0.4, *p* = 0.001), Δ CRR (beta −1.3, *p*< 0.001), and Δ LDLc (beta 0.6, *p* = 0.01).

The stepwise regression model identified the factors most likely to change the GLS. Δ CRR and Δ FPG play an important role in these changes, reinforcing the importance of lipid management and glycemic control in patients with T2DM in stages A and B of HF. However, we found an unexpected inverse proportional association between Δ GLS and ΔLDLc, suggesting that as LDLc increases, GLS also improves.

## 4. Discussion

The present study brings evidence that oral semaglutide, as an add-on to the standard of care, exerts pleiotropic effects on subclinical changes in T2DM patients in stages A and B of HF after one year of treatment. One of the most significant changes observed was the improvement in GLS, demonstrating a reversal of myocardial dysfunction. This study included 50 patients with a mean age of 58.8 ± 10.4 years, with a diabetes duration of a median of 7 years, and with different degrees of obesity at the study enrollment. Patients had a mean LVEF of 56.6 ± 6.0% at baseline and a median GLS of −16 (11;20)%. Also, patients presented a significant decrease in FPG, TyG, and HbA1c, suggesting improved insulin sensitivity. While LDLc and HDLc did not reach individual statistical significance, overall, the TC improved significantly. Moreover, the atherogenic indexes CRR and AC significantly decreased. Although LVEF did not improve in dynamics, the GLS changes were significant.

The results obtained are in line with the literature data, according to which treatment with glucagon-like peptide-1 receptor agonist (GLP-1RA) can restore cardiac metabolic flexibility by reducing body weight, lowering blood glucose, and improving insulin sensitivity, increasing glucose uptake and utilization. GLP-1 receptors (GLP1-R) are known to be expressed on endothelial and smooth muscle cells in blood vessels and atrial and ventricular cardiomyocytes, suggesting that they may directly affect cardiac tissue. Possible mechanisms underlying clinical cardiovascular benefits may be anti-inflammatory, anti-atherosclerotic, vasodilatory, and other hemodynamic actions. In vitro studies on blood vessels indicate that GLP1-RA inhibits the proliferation of vascular smooth muscle cells, reduces reactive oxygen species, and enhances nitric oxide levels in vascular endothelial cells [26,27,28]. Also, in a murine model of Heart Failure with Preserved Ejection Fraction (HFpEF), it was identified that semaglutide decreased intramyocardial lipid accumulation, improved adenosine triphosphate availability, and reduced both cardiac hypertrophy and fibrosis, while maintaining mitochondrial structure and function [29]. In our study, the patients who remained on treatment with oral semaglutide at the maximum tolerated dose at the follow-up presented a mean decrease in weight of −5.6 kg, as well as a mean decrease in abdominal waist of −4.7 cm, resulting in a significantly lower BMI by −2 kg/m^2^, and a lower conicity index and LAP. Also, we observed a reduction in EAT, which underscores the interrelation between metabolic status and cardiac function in T2DM patients. Our regression analysis identified several potential predictors of GLS improvement among EAT, LAP, abdominal waist, FPG, HbA1c, CRR, and LDLc.

The GLS improvement demonstrated in our study (−0.7%) is clinically significant and extremely valuable, considering the sensitivity of this marker to identifying subclinical systolic dysfunction. We demonstrated that CRR and FPG play an important role in GLS changes, reinforcing the importance of lipid management and glycemic control in patients with T2DM who are in stages A and B of HF. It is important to comment on the positive correlation between changes in GLS and LDLc, which may contradict conventional beliefs. This observation may reflect a shift towards less atherogenic LDL particles, but further investigation is needed to understand changes in lipid profiling, including LDL particle size and number, apolipoprotein B levels, and other markers of atherogenicity after oral semaglutide administration. GLS is a known marker of subclinical systolic dysfunction and has been shown to predict future cardiovascular events [6]. An improved GLS value correlates with improved myocardial contractility and a decreased risk of developing HF progression. As a result, fewer hospitalizations and improved long-term survival have been documented. Studies like SUSTAIN-6 and SELECT have shown that GLP-1 receptor agonists reduce major adverse cardiovascular events, including HF hospitalizations. These findings support the hypothesis that improvements in GLS, as observed in our study, could translate into reduced HF risk and improved long-term cardiovascular outcomes in T2DM patients. Our study extends the findings from SUSTAIN-6 and SELECT studies about reduced major adverse cardiovascular events after treatment with semaglutide, suggesting some mechanistic insights of oral semaglutide in improving subclinical cardiac [30,31].

Similarly, a study conducted by G Armentar et al. demonstrated that in patients with uncontrolled T2DM and obesity who were treated for 6 months with semaglutide, GLS, and global myocardial work efficiency (GWE) were improved. The observed improvements could have been justified by a reduction in inflammatory markers, oxidative stress, and platelet activation, alongside favorable metabolic changes, which protect coronary microcirculation and positively affect myocardial contractility [32]. Another pilot study by Paolo Basile et al. showed that six months of treatment with GLP-1 RA (dulaglutide or semaglutide) led to an improvement in GLS in subjects with T2DM and with a high/very high risk of atherosclerotic cardiovascular disease (ASCVD) or with established ASCVD [28].

Similar evidence is provided by another study conducted by Melissa Leung et al., in which similar echocardiographic methods were used on 105 patients with T2DM and poor glycemic control. The study demonstrated that improved glycemic control over 12 months improved LV systolic and diastolic function. They observed a progressively greater improvement in GLS as patients achieved a lower HbA1c. However, the decrease in BMI and the use of metformin were additional independent predictors of GLS improvement [33]. In our study, BMI and weight reached only marginal statistical significance, with the abdominal waist being more strongly associated with changes in GLS, suggesting that abdominal adiposity may be linked with these subclinical changes in stage A and B HF. Weight reduction is associated with decreased cardiac workload, reduced left ventricular mass, and improved diastolic function and hemodynamics. Visceral fat is considered a metabolically active organ due to the secretion of various proinflammatory cytokines; reducing visceral adipose tissue will result in lower systemic inflammation and improved endothelial function. Semaglutide’s effects on glycemic control and lipid metabolism reduce the metabolic stress burden on the heart. These combined effects of GLP-1 receptor activation with oral semaglutide could improve systolic and diastolic performance.

The present study’s findings on the effects of oral semaglutide on GLS after one year of therapy in patients with diabetes who are at risk of HF, particularly those with metabolic risk factors who are at risk of HF (stage A HF), align with the echocardiography substudy of the STEP-HFpEF Program [34], where semaglutide improved left atrial remodeling and right ventricular enlargement compared with a placebo, independently of weight loss effects on E-wave velocity and E/e′. Although our findings apply predominantly to stage A HF patients, they may also be relevant to early-stage B HF, representing the pre-symptomatic phases of HF according to the 2022 AHA/ACC/HFSA Guidelines for the Management of Heart Failure [35]. While we focused on the subclinical cardiac benefits of oral semaglutide, the STEP-HFpEF Program proved a reduction in combined CV death and worsening HF events. Our study’s improvements in subclinical parameters suggest clinical benefits if oral semaglutide is administered from stage A of HF.

We admit several limitations of our study. The relatively small sample size and single-center design may limit the generalizability of our findings. Also, the lack of detailed lipid profiling beyond standard lipid particles limits our understanding of the mechanisms underlying our results. The observed correlations may represent a transitional state in lipid metabolism during treatment with semaglutide. Also, statin therapy could be a confounding factor that may have influenced the association between LDLc and GLS. Since all the patients were on statins, we could not compare outcomes between statin users and non-users. While we controlled for several confounders in our analyses, the study was not explicitly designed to investigate the interaction between statin therapy and the relationship between LDLc and GLS. Although our study did not stratify by obesity class or explicitly examine the dose–response relationship, the findings from STEP-HFpEF showed consistency across obesity grades. They demonstrated that the magnitude of semaglutide benefits was directly associated with the extent of weight loss. The study also presents several strengths that should be acknowledged, such as the prospective study design that allowed for assessments of complex cardiac and metabolic changes over a relatively long period, and advanced echocardiographic techniques like speckle-tracking for GLS measurement over time. Also, the study focused on subclinical cardiac dysfunction in T2DM patients, a topic of increased interest for early intervention. During our study, 22 patients were lost to follow-up or did not tolerate oral semaglutide until the end of the study, which may limit the generalizability of our findings. Despite this limitation, the remaining cohort provided valuable insights into the effects of oral semaglutide on subclinical cardiac changes in T2DM patients with stage A or stage B HF. Our study focused on a predominantly European population with overweight or obesity, which may limit the direct applicability of our findings to diverse ethnic groups or normal-weight individuals. Future studies should confirm these findings in diverse populations to enhance the generalizability of our results.

## 5. Conclusions

After one year of therapy, oral semaglutide improves GLS in patients with T2DM and stage A HF (patients at risk of HF) or stage B HF (pre-HF). TC/HDLc, LDLc, and FPG are potential strong predictors of GLS changes during oral semaglutide treatment. An improved TC/HDL ratio (CRR) and improved FPG are associated with attenuated GLS. Although, previously, low LDLc has usually been associated with reduced CV risk, our study showed that increases in LDLc were related to improvements in GLS. This finding may reflect a shift toward larger LDLc particles that are less atherogenic. However, future studies, including more detailed lipid profiling, are needed to understand our findings fully. Further research is required to understand the mechanisms underlying these associations between GLS and metabolic biomarkers and their clinical implications.

## Figures and Tables

**Table 1:** Comparison of baseline general characteristics by gender.

Variable	Overall	Women	Men	*p* for Gender
Age (years) ^b^	58.8 ± 10.4	59.3 ± 11.9	58.1 ± 8.6	0.6
Weight (kg) ^b^	98.7 ± 20.5	92.8 ± 19.2	105.6 ± 19.3	0.02
Height (cm) ^b^	169.4 ± 9.4	163.1 ± 6.6	176.8 ± 6.3	<0.001
BMI (kg/m^2^) ^b^	34.3 ± 6.4	34.8 ± 7.2	33.7 ± 5.4	0.5
Waist (cm) ^b^	116.4 ± 11.7	113.5 ± 12.5	120.0 ± 9.9	0.05
Diabetes duration (years) ^a^	7.0 (0.0;21.0)	7.0 (0.0;20.0)	5.0 (0.0;21.0)	0.4
Conicity index ^b^	140.4 ± 6.2	138.8 ± 0.3	142.4 ± 0.9	0.04
LAP ^b^	108.2 ± 59.8	112.7 ± 51.5	102.7 ± 69.6	0.5
TG (mg/dL) ^b^	154.5 (54.0;597.0)	181.8 ± 78.8	168.5 ± 125.5	0.6
TC (mg/dL) ^b^	181.5 ± 48.4	196.5 ± 50.0	164.5 ± 41.2	0.01
AIP ^b^	0.1 ± 0.2	0.1 ± 0.2	0.1 ± 0.0	0.7
CRR ^a^	3.7 (2.0;8.1)	3.7 (2.0;8.1)	3.7 (2.3;7.4)	0.6
AC ^a^	2.7 (1.0;7.1)	2.7 (1.0;7.1)	2.7 (1.3;6.4)	0.6
HDLc (mg/dL) ^b^	46.7 ± 12.8	51.5 ± 14.0	41.2 ± 8.8	0.004
LDLc (mg/dL) ^b^	107.6 ± 38.0	117.1 ± 40.1	95.5 ± 32.1	0.05
VAI ^a^	1.9 (0.5;10)	2.1 (0.5;6.3)	2.8 (0.8;10.0)	0.9
Uric acid (mg/dL) ^b^	5.5 ± 1.3	5.4 ± 1.4	5.6 ± 1.1	0.5
TyG ^b^	5.0 ± 0.3	5.0 ± 0.3	5.0 ± 0.4	0.8
FPG (mg/dL) ^b^	153.0 (84.0;294.0)	173.4 ± 0.2	155.6 ± 0.0	0.2
HbA1c (%) ^b^	7.9 ± 1.4	7.9 ± 0.2	7.9 ± 0.3	0.9
eGFR (mL/min) ^b^	90.6 ± 18.0	87.2 ± 0.5	94.6 ± 0.9	0.1
UACr (mg/g) ^a^	7.8 (2.3;655.9)	11.1 (3.7;122.8)	4.8 (2.3;655.9)	0.4
NLR ^a^	1.9 (0.8;5.8)	1.873 (0.9;4.2)	2.1 (0.8;5.8)	0.4
PLR ^a^	103.2 (58.1;213)	103.8 (58.1;213.0)	102.3 (58.6;196.6)	0.3
SII (×10^3^) ^a^	478.1 (147.2;1682.8)	459.2 (209.0;1682.8)	496.9 (147.2;1374.5)	0.5
RV (cm) ^b^	2.9 ± 0.3	2.7 ± 0.1	3.0 ± 0.2	0.002
IVS (cm) ^b^	1.1 ± 0.1	1.1 ± 0.3	1.2 ± 0.1	0.2
LVEDD (cm) ^b^	4.3 ± 0.5	4.2 ± 0.6	4.5 ± 0.8	0.08
PWT (cm) ^b^	1.1 ± 0.1	1.1 ± 0.4	1.1 ± 0.6	0.1
Ascending AO ^b^ (cm)	2.8 ± 0.4	2.7 ± 0.1	3.0 ± 0.4	0.01
LA ^b^ (cm)	3.7 ± 0.5	3.6 ± 0.7	3.7 ± 0.2	0.4
AOx (cm) ^b^	2.7 ± 0.3	2.7 ± 0.3	2.6 ± 0.7	0.8
EAT ^a^ (mm)	4 (0.4;9.3)	4 (0.4;9.10)	4 (2.6;9.3)	0.2
PV PA (cm/s) ^a^	0.8 (0.6;1.5)	1.2 (0.8;3.2)	1.1 (0.7;2.4)	0.5
PAAT (ms)^b^	120 (92;150)	122.5 ± 16.7	121.2 ± 16.4	0.8
LVEDV (mL)^b^	109.2 ± 27.5	99.6 ± 22.0	121.1 ± 29.4	0.005
LAv (mL) ^b^	49.1 ± 16.03	47.2 ± 14.0	51.2 ± 18.1	0.3
LVEF (%)^a^	55.0 (45.0;70.0)	56.2 ± 6.4	56.2 ± 5.7	0.7
MV E-wave Vmax ^b^ (cm/s)	64.3 ± 14.4	66.5 ± 13.8	61.8 ± 15.1	0.2
MV A-wave Vmax ^b^ (cm/s)	80.3 ± 16.7	83.2 ± 15.3	77.0 ± 18.0	0.2
E/A ^a^	0.7 (0.3;1.6)	0.7 (0.5;1.5)	0.7 (0.3;1.6)	0.8
DTE (ms) ^b^	218.2 ± 40.9	222.1 ± 43.3	213.5 ± 38.2	0.4
PV AO (cm/s)^a^	1.2 (0.7;3.2)	1.1 (0.8;2.4)	1.125 (0.1;2.3)	0.1
MV e′ septum ^a^ (cm/s)	8.0 (4.0;16.0)	8.0(5.0;15.0)	7.0 (4.0;16.0)	0.2
MV a′ septum ^b^ (cm/s)	10.2 ± 2.2	9.8 ± 1.9	10.8 ± 2.5	0.1
MV s′ septum ^a^ (cm/s)	7.0 (4.0;12.0)	7.0 (4.0;12.0)	7.0 (5.0;10.0)	0.5
MV e′ lateral wall ^a^ (cm/s)	9.0 (5.0;15.0)	9.4 ± 2.7	9.7 ± 2.9	0.7
MV a′ lateral wall ^a^ (cm/s)	12.0 (5.0;17.0)	11.5 ± 2.5	11.5 ± 3.4	0.9
MV s′ lateral wall ^a^ (cm/s)	8.0 (5.0;16.0)	8.0 (6.0;12.0)	9.0 (5.0;16.0)	0.2
Average E′ (cm/s) **^a^**	8.6 (5;14)	9.1 ± 1.7	8.6 ± 2.9	0.4
average E/e′ ^b^	7.3 (4.2;13.3)	7.6 ± 1.8	7.6 ± 2.0	0.9
TAPSE ^b^ (cm)	2.4 ± 0.4	2.4 ± 0.3	2.5 ± 0.5	0.7
GLS ^a^ (%)	−16.0 (11.0;20)	−16.2 ± 1.8	−16.0 ± 1.3	0.7

Abbreviations: BMI, body mass index; LAP, lipid accumulation product; TG, triglycerides; TC, total cholesterol; AIP, atherogenic index of plasma; CRR, cardiac risk ratio; AC, atherogenic coefficient; HDLc, high-density lipoprotein cholesterol; LDLc, low-density lipoprotein cholesterol; VAI, visceral adiposity index; TyG, product of triglycerides and glucose; FPG, fasting plasma glucose; HbA1c, glycated hemoglobin A1c; eGFR, estimated glomerular filtration rate; UACr, urinary albumin/creatinine ratio; NLR, neutrophil-to-lymphocyte ratio; PLR, platelet-to-lymphocyte ratio; SII, systemic immune-inflammation index; RV, right ventricle; IVS, interventricular septum; LVEDD, end-diastolic diameter of the left ventricle; PWT, posterior wall thickness; LA, left atrium; LAv, left atrium volume; AOx, aortic cross; PV PA, pulmonary artery peak velocity; LVEDV, left ventricle end-diastolic volume; MV, mitral valve; AO, aorta; EAT, epicardiac adipose tissue; PAAT, pulmonary artery acceleration time; DTE, E deceleration time; PV Ao, peak velocity aorta; TAPSE, tricuspid annular plane systolic excursion; GLS, global longitudinal strain; LVEF, left ventricle ejection fraction; ^a^ Mann–Whitney test, ^b^ *t*-test for gender comparison.

**Table 2:** The dynamics of the studied parameters after one year of oral semaglutide.

Variable	Before Oral Semaglutide Add-On	After One Year of Semaglutide Add-On	Paired Differences	*p*
Weight (kg) ^b^	99.2 ± 21.5	93.5 ± 21.0	−5.6 ± 6.0	<0.0001
BMI (kg/m^2^) ^b^	35.0 ± 6.5	32.9 ± 6.1	−2.0 ± 2.0	<0.0001
Waist (cm) ^b^	115.8 ± 10.9	111.1 ± 9.5	−4.7 ± 7.6	0.0002
Conicity index ^b^	140.0 ± 6.1	1.3 ± 0.3	−138.7 ± 6.1	<0.0001
LAP ^b^	105.3 ± 57.8	83.0 ± 41.7	−22.3 ± 66.6	0.03
TG (mg/dL) ^b^	177.1 ± 102.6	155.4 ± 80.9	−21.6 ± 111.2	0.1
TC (mg/dL) ^b^	180.2 ± 51.5	158.2 ± 42.5	−21.9	0.01
AIP ^b^	0.1 ± 0.3	0.1 ± 0.2	−0.06 ± 0.2	0.1
CRR ^a^	3.6	3.1	−0.4	0.001
AC ^a^	2.6	2.1	−0.4	0.001
HDLc (mg/dL) ^b^	47.4 ± 15.1	50.4 ± 15.1	2.9 ± 9.5	0.08
LDLc (mg/dL) ^b^	103.3 ± 37.2	92.2 ± 30.0	−11.1 ± 43.6	0.1
VAI ^a^	2.0	1.7	−0.2	0.1
Uric acid (mg/dL) ^b^	5.6 ± 1.4	5.6 ± 1.3	−0.005 ± 0.7	0.9
TyG ^b^	5.0 ± 0.3	4.9 ± 0.2	−0.1 ± 0.3	0.001
FPG (mg/dL) ^b^	165.5 ± 50.5	137.8 ± 30.2	−27.7 ± 45.9	0.0001
HbA1c (%) ^b^	7.9 ± 1.6	6.8 ± 1.0	−1.1 ± 1.5	0.0009
eGFR (mL/min) ^b^	90.2 ± 18.7	87.4 ± 20.3	−2.7 ± 14.2	0.2
UACr (mg/g) ^a^	11.1	7.3	−0.7	0.1
NLR ^a^	1.9	1.9	0.0	0.4
PLR ^a^	110.0	104.2	−5.9	0.5
SII T0 (×10^3^) ^a^	502.0	455.6	8.4	0.9
RV (cm) ^b^	2.9 ± 0.3	2.9 ± 0.3	0.08 ± 0.3	0.1
IVS (cm) ^b^	1.1 ± 0.1	1.4 ± 1.5	0.2 ± 1.5	0.2
LVEDD (cm) ^b^	4.3 ± 0.5	4.3 ± 0.4	−0.03 ± 0.3	0.5
PWT (cm) ^b^	1.1 ± 0.1	1.3 ± 1.5	0.2 ± 1.5	0.2
Ascending AO ^b^ (cm)	2.8 ± 0.4	3.0 ± 0.4	0.1 ± 0.3	0.02
LA ^b^ (cm)	3.7 ± 0.5	3.6 ± 0.4	−0.1 ± 0.4	0.1
AOx (cm) ^b^	2.7 ± 0.3	2.7 ± 0.3	0.0 ± 0.2	0.5
EAT ^a^ (mm)	4.0	3.3	−0.3	0.0005
PV PA (cm/s) ^b^	0.8 ± 0.1	1.0 ± 1.4	0.2 ± 1.4	0.3
PAAT (ms) ^b^	121.9 ± 16.4	117.2 ± 15.3	−4.7 ± 16.0	0.05
LVEDV (mL) ^b^	109.2 ± 27.5	102.9 ± 20.8	−6.3 ± 22.7	0.05
LAv (mL) ^b^	49.1 ± 16.0	48.8 ± 13.8	−0.2 ± 12.1	0.8
LVEF (%) ^b^	56.6 ± 6.0	56.5 ± 5.8	−0.1 ± 4.5	0.8
MV E-wave Vmax ^b^ (cm/s)	64.9 ± 14.1	63.7 ± 16.1	−1.2 ± 16.6	0.6
MV A-wave Vmax ^b^ (cm/s)	79.8 ± 16.3	80.6 ± 17.3	0.8 ± 16.6	0.7
E/A ^a^	0.7	0.7	0.0	0.7
DTE (ms)^b^	218.0 ± 41.3	217.8 ± 49.4	−0.1 ± 45.3	0.9
PV AO (cm/s)^b^	1.2 ± 0.3	1.1 ± 0.3	−0.0 ± 0.3	0.1
MV e′ septum ^a^ (cm/s)	8.0	7.0	−1.0	0.03
MV a′ septum ^b^ (cm/s)	10.3 ± 2.2	9.9 ± 2.2	−0.4 ± 1.9	0.1
MV s′ septum ^a^ (cm/s)	7.0	7.0	0.0	0.4
MV e′ lateral wall ^b^ (cm/s)	9.6 ± 2.7	10.0 ± 3.1	0.3 ± 2.2	0.2
MV a′ lateral wall ^b^ (cm/s)	11.6 ± 2.9	12.0 ± 2.9	0.4 ± 3.5	0.3
MV s′ lateral wall ^a^ (cm/s)	8.9 ± 2.4	9.0 ± 2.1	0.0 ± 1.7	0.9
Average E′ (cm/s) ^b^	8.9 ± 2.3	8.7 ± 2.3	−0.2 ± 1.9	0.4
Average E/e′ ^b^	7.6 ± 1.9	7.6 ± 2.2	−0.0 ± 2.2	0.9
TAPSE ^b^ (cm)	2.4 ± 0.4	2.5 ± 0.4	0.0 ± 0.2	0.1
GLS ^b^ (%)	−16.1 ± 1.6	−16.8 ± 1.9	0.7 ± 0.8	<0.0001

Abbreviations: BMI, body mass index; LAP, lipid accumulation product; TG, triglycerides; TC, total cholesterol; AIP, atherogenic index of plasma; CRR, cardiac risk ratio; AC, atherogenic coefficient; HDLc, high-density lipoprotein cholesterol; LDLc, low-density lipoprotein cholesterol; VAI, visceral adiposity index; TyG, product of triglycerides and glucose; FPG, fasting plasma glucose; HbA1c, glycated hemoglobin A1c; eGFR, estimated glomerular filtration rate; UACr, urinary albumin/creatinine ratio; NLR, neutrophil-to-lymphocyte ratio; PLR, platelet-to-lymphocyte ratio; SII, systemic immune-inflammation index; RV, right ventricle; IVS, interventricular septum; LVEDD, left ventricle end-diastolic diameter; PWT, posterior wall thickness; LA, left atrium; LAv, left atrium volume; AOx, aortic cross, PV PA, pulmonary artery peak velocity; LVEDV, left ventricle end-diastolic volume; MV, mitral valve; AO, aorta; EAT, epicardiac adipose tissue; PAAT, pulmonary artery acceleration time; DT, deceleration time; PV Ao, peak velocity aorta; TAPSE, tricuspid annular plane systolic excursion; GLS, global longitudinal strain; LVEF, left ventricle ejection fraction; ^a^ Mann–Whitney test, ^b^ *t*-test.

**Table 3:** Correlation of Δ GLS with differences in dynamics of anthropometric and metabolic parameters in patients with type 2 diabetes treated for one year with oral semaglutide.

			Pearson		Spearman	
			r	*p*	rho	*p*
Δ GLS	-	Δ Weight (kg)	−0.0	0.6	0.0	0.9
	-	Δ BMI (kg/m^2^)	0.0	0.8	0.0	0.8
	-	Δ Waist (cm)	0.0	0.7	0.0	0.6
	-	Δ Conicity index	0.0	0.6	−0.0	0.8
	-	Δ VAI	−0.3	0.05	−0.3	0.04
	-	Δ FPG (mg/dL)	−0.2	0.09	−0.3	0.009
	-	Δ HbA1c (%)	−0.0	0.9	−0.1	0.3
	-	Δ Uric acid (mg/dL)	−0.0	0.6	−0.2	0.1
	-	Δ TG (mg/dL)	−0.4	0.005	−0.4	0.004
	-	Δ LDLc (mg/dL)	−0.6	<0.001	−0.4	0.009
	-	Δ HDLc (mg/dL)	0.0	0.9	0.0	0.7
	-	Δ TC (mg/dL)	−0.3	0.01	−0.2	0.1
	-	Δ eGFR (mL/min)	0.0	0.9	−0.0	0.8
	-	Δ UACr (mg/g)	−0.0	0.7	−0.3	0.1
	-	Δ TyG	−0.2	0.09	−0.3	0.02
	-	Δ LAP	−0.3	0.03	−0.3	0.03
	-	Δ SII (×10^3^)	−0.0	0.9	0.0	0.7
	-	Δ CRR	−0.5	0.003	−0.3	0.03
	-	Δ AC	−0.5	0.003	−0.3	0.03
	-	Δ AIP	−0.4	0.02	−0.4	0.01

Abbreviations: BMI, body mass index; LAP, lipid accumulation product; TG, triglycerides; TC, total cholesterol; AIP, atherogenic index of plasma; CRR, cardiac risk ratio; AC, atherogenic coefficient; HDLc, high-density lipoprotein cholesterol; LDLc, low-density lipoprotein cholesterol; VAI, visceral adiposity index; TyG, product of triglycerides and glucose; FPG, fasting plasma glucose; HbA1c, glycated hemoglobin A1c; eGFR, estimated glomerular filtration rate; UACr, urinary albumin/creatinine ratio; NLR, neutrophil-to-lymphocyte ratio; PLR, platelet-to-lymphocyte ratio; SII, systemic immune-inflammation index.

**Table 4:** Correlation of Δ GLS with differences in dynamics of speckle-tracking echocardiography parameters in patients with type 2 diabetes treated for one year with oral semaglutide.

			Pearson		Spearman	
			r	*p*	rho	*p*
Δ GLS	-	Δ EAT (mm)	−0.0	0.6	−0.1	0.4
	-	Δ E/A	0.0	0.8	0.1	0.2
	-	Δ E (cm/s)	−0.0	0.9	0.0	0.6
	-	Δ A (cm/s)	−0.0	0.8	−0.1	0.3
	-	Δ average E/e′	0.0	0.6	0.1	0.2
	-	Δ LVEF (%)	0.1	0.4	−0.0	0.5
	-	Δ LVEDV (mL)	−0.0	0.9	−0.0	0.6
	-	Δ RV (cm)	0.0	0.8	0.0	0.9
	-	Δ LAv (mL)	−0.0	0.9	0.0	0.7
	-	Δ s′ septal wall (cm/s)	−0.1	0.4	−0.2	0.1
	-	Δ s′ lateral wall (cm/s)	−0.1	0.2	−0.3	0.02
	-	Δ average E′	−0.1	0.5	−0.2	0.1
	-	Δ e′ lateral wall (cm/s)	−0.1	0.2	−0.0	0.6
	-	Δ e′ septal wall (cm/s)	−0.2	0.1	−0.4	0.005
	-	Δ a′ lateral wall (cm/s)	−0.1	0.2	−0.1	0.4
	-	Δ a′ septal wall (cm/s)	−0.0	0.8	−0.0	0.6
	-	Δ PV PA (cm/s)	−0.0	0.9	−0.4	0.01
	-	Δ PV Ao (cm/s)	−0.2	0.07	−0.1	0.2
	-	Δ Ascending AO (cm)	−0.0	0.5	0.0	0.7
	-	Δ AOx (cm)	0.1	0.5	0.2	0.1
	-	Δ LA (cm)	−0.0	0.5	−0.1	0.3
	-	Δ LVEDD (cm)	0.1	0.3	0.2	0.1
	-	Δ PWT (cm)	−0.0	0.5	−0.1	0.2
	-	Δ IVS (cm)	−0.0	0.7	−0.1	0.3
	-	Δ PAAT	−0.1	0.4	−0.1	0.3
	-	Δ TAPSE	−0.1	0.3	−0.1	0.3
	-	Δ DTE	0.1	0.4	0.1	0.2

Abbreviations: Δ, the difference between baseline and one-year follow-up; EAT, epicardiac adipose tissue; RV, right ventricle; IVS, interventricular septum; LVEDD, left ventricular end-diastolic diameter; PWT, posterior wall thickness; LA, left atrium; LAv, left atrium volume; AOx, aortic cross; PV PA, pulmonary artery peak velocity; LVEDV, left ventricle end-diastolic volume; MV, mitral valve; AO, aorta; PAAT, pulmonary artery acceleration time; DTE, E deceleration time; PV Ao, peak velocity aorta; TAPSE, tricuspid annular plane systolic excursion; GLS, global longitudinal strain; LVEF, left ventricle ejection fraction.

**Table 5:** Linear regression analysis in a backward manner for predicting Δ GLS in patients with type 2 diabetes treated one year with oral semaglutide, an add-on to the standard of care.

Model		Unstandardized Coefficient	Standard Error	Beta	t	*p*-Value
M_0_	(Intercept)	32.2	17.0		1.8	0.3
	Δ TyG	−0.0	0.7	0.0	−0.0	0.9
	Δ FPG (mg/dL)	−0.0	0.0	−0.6	−1.6	0.3
	Δ BMI (kg/m^2^)	−1.2	1.1	−2.3	−1.0	0.4
	Δ Weight (kg)	0.3	0.3	1.8	0.9	0.5
	Δ Conicity index	−0.2	0.1	−1.0	−1.8	0.3
	Δ CRR	−2.2	0.4	−2.3	−4.5	0.1
	Δ Waist (cm)	0.1	0.0	1.0	1.8	0.3
	Δ VAI	0.0	1.0	0.0	0.0	0.9
	Δ SII (×10^3^)	0.0	0.0	0.0	0.2	0.8
	Δ LAP	−0.0	0.0	−1.6	−1.9	0.2
	Δ EAT (mm)	0.2	0.2	0.3	0.9	0.5
	Δ HbA1c (%)	0.1	0.1	0.4	1.6	0.3
	Δ HDLc (mg/dL)	−0.1	0.0	−1.2	−2.1	0.2
	Δ LDLc (mg/dL)	0.0	0.0	1.0	1.8	0.3
	Δ LVEF (%)	−0.0	0.1	−0.3	−0.7	0.5
	Δ PWT (cm)	4.2	4.7	0.6	0.8	0.5
	Δ s′ septal	0.3	0.3	0.5	1.1	0.4
	Δ s′ lateral	−0.4	0.3	−0.7	−1.5	0.3
	Δ IVS (cm)	−6.1	6.4	−0.7	−0.9	0.5
	Δ TG (mg/dL)	0.0	0.0	2.5	1.8	0.3
M_1_	(Intercept)	32.2	11.0		2.9	0.1
	Δ FPG (mg/dL)	−0.0	0.0	−0.6	−2.6	0.1
	Δ BMI (kg/m^2^)	−1.2	0.8	−2.3	−1.5	0.2
	Δ Weight (kg)	0.3	0.2	1.8	1.3	0.3
	Δ Conicity index	−0.2	0.0	−1.0	−2.9	0.1
	Δ CRR	−2.2	0.3	−2.3	−6.4	0.02
	Δ Waist (cm)	0.1	0.0	1.0	2.6	0.1
	Δ VAI	0.0	0.6	0.0	0.0	0.9
	Δ SII (×10^3^)	0.0	0.0	0.0	0.3	0.7
	Δ LAP	−0.0	0.0	−1.7	−2.8	0.1
	Δ EAT (mm)	0.2	0.1	0.3	1.4	0.2
	Δ HbA1c (%)	0.1	0.0	0.4	2.2	0.1
	Δ HDLc (mg/dL)	−0.1	0.0	−1.2	−3.0	0.09
	Δ LDLc (mg/dL)	0.0	0.0	1.0	2.8	0.1
	Δ LVEF (%)	−0.0	0.0	−0.3	−1.0	0.3
	Δ PWT (cm)	4.2	3.1	0.6	1.3	0.3
	Δ s′ septal	0.3	0.2	0.5	1.6	0.2
	Δ s′ lateral	−0.4	0.2	−0.7	−2.2	0.1
	Δ IVS (cm)	−6.1	4.4	−0.7	−1.3	0.3
	Δ TG (mg/dL)	0.0	0.0	2.5	2.6	0.1
M_2_	(Intercept)	32.1	8.7		3.6	0.03
	Δ FPG (mg/dL)	−0.0	0.0	−0.6	−3.2	0.04
	Δ BMI (kg/m^2^)	−1.1	0.5	−2.3	−2.0	0.1
	Δ Weight (kg)	0.3	0.1	1.7	1.8	0.1
	Δ Conicity index	−0.2	0.0	−1.0	−3.6	0.03
	Δ CRR	−2.2	0.2	−2.3	−7.9	0.004
	Δ Waist (cm)	0.1	0.0	1.0	3.2	0.04
	Δ SII (×10^3^)	0.0	0.0	0.1	0.4	0.6
	Δ LAP	−0.0	0.0	−1.6	−3.8	0.03
	Δ EAT (mm)	0.2	0.1	0.3	1.7	0.1
	Δ HbA1c (%)	0.1	0.0	0.4	2.8	0.06
	Δ HDLc (mg/dL)	−0.1	0.0	−1.2	−4.4	0.02
	Δ LDLc (mg/dL)	0.0	0.0	1.0	4.0	0.02
	Δ LVEF (%)	−0.0	0.0	−0.3	−1.4	0.2
	Δ PWT (cm)	4.1	1.9	0.6	2.0	0.1
	Δ s′ septal	0.3	0.1	0.5	2.0	0.1
	Δ s′ lateral	−0.4	0.1	−0.7	−2.7	0.07
	Δ IVS (cm)	−6.0	3.4	−0.7	−1.7	0.1
	Δ TG (mg/dL)	0.0	0.0	2.5	4.2	0.02
M_3_	(Intercept)	33.4	7.4		4.5	0.01
	Δ FPG (mg/dL)	−0.0	0.0	−0.7	−4.4	0.01
	Δ BMI (kg/m^2^)	−1.3	0.4	−2.5	−2.8	0.04
	Δ Weight (kg)	0.3	0.1	1.9	2.4	0.06
	Δ Conicity index	−0.2	0.0	−1.1	−4.5	0.01
	Δ CRR	−2.2	0.2	−2.3	−8.8	<0.001
	Δ Waist (cm)	0.1	0.0	1.1	4.6	0.009
	Δ LAP	−0.0	0.0	−1.7	−4.7	0.009
	Δ EAT (mm)	0.2	0.0	0.4	2.7	0.05
	Δ HbA1c (%)	0.1	0.0	0.4	4.1	0.01
	Δ HDLc (mg/dL)	−0.1	0.0	−1.2	−5.1	0.007
	Δ LDLc (mg/dL)	0.0	0.0	1.0	4.6	0.01
	Δ LVEF (%)	−0.1	0.0	−0.4	−2.6	0.06
	Δ PWT (cm)	4.7	1.3	0.7	3.3	0.02
	Δ s′ septal	0.3	0.1	0.6	3.1	0.03
	Δ s′ lateral	−0.5	0.1	−0.7	−4.5	0.01
	Δ IVS (cm)	−7.2	2.0	−0.8	−3.5	0.02
	Δ TG (mg/dL)	0.0	0.0	2.6	5.3	0.006

Statistical significance: *p* for M_0_ = 0.3, adjusted R^2^ = 0.7, RMSE = 0.5; *p* for M_1_ = 0.1, adjusted R^2^ = 0.8, RMSE = 0.4; *p* for M_2_ = 0.03, adjusted R^2^ = 0.9, RMSE = 0.3; *p* for M_3_ = 0.008, adjusted R^2^ = 0.9, RMSE = 0.3. Abbreviations: BMI, body mass index; LAP, lipid accumulation product; TG, triglycerides; TC, total cholesterol; AIP, atherogenic index of plasma; CRR, cardiac risk ratio; AC, atherogenic coefficient; HDLc, high-density lipoprotein cholesterol; LDLc, low-density lipoprotein cholesterol; VAI, visceral adiposity index; TyG, product of triglycerides and glucose; FPG, fasting plasma glucose; HbA1c, glycated hemoglobin A1c; eGFR, estimated glomerular filtration rate; UACr, urinary albumin/creatinine ratio; NLR, neutrophil-to-lymphocyte ratio; PLR, platelet-to-lymphocyte ratio; SII, systemic immune-inflammation index; RV, right ventricle; IVS, interventricular septum; LVEDD, left ventricle end-diastolic diameter; PWT, posterior wall thickness; LA, left atrium; LAv, left atrium volume; AOx, aortic cross; PV PA, pulmonary artery peak velocity; LVEDV, left ventricle end-diastolic volume; MV, mitral valve; AO, aorta; EAT, epicardiac adipose tissue; PAAT, pulmonary artery acceleration time; DT, deceleration time; PV Ao, peak velocity aorta; TAPSE, tricuspid annular plane systolic excursion; GLS, global longitudinal strain; LVEF, left ventricle ejection fraction.

**Table 6:** Linear regression analysis in a stepwise manner for predicting Δ GLS in patients with type 2 diabetes treated for one year with oral semaglutide, an add-on to the standard of care.

Coefficients						
Model		Unstandardized Coefficient	Standard Error	Beta	t	*p*-Value
M_0_	(Intercept)	−0.7	0.2		−3.1	0.005
M_1_	(Intercept)	−0.3	0.1		−2.3	0.03
	Δ CRR	−0.7	0.1	−0.8	−6.1	<0.001
M_2_	(Intercept)	−0.2	0.1		−1.3	0.1
	Δ CRR	−0.7	0.1	−0.7	−6.7	<0.001
	Δ FPG (mg/dL)	−0.0	0.0	−0.2	−2.5	0.02
M_3_	(Intercept)	−0.0	0.1		−0.1	0.9
	Δ CRR	−1.2	0.2	−1.3	−5.8	<0.001
	Δ FPG (mg/dL)	−0.0	0.0	−0.4	−3.8	0.001
	ΔLDLc (mg/dL)	0.0	0.0	0.6	2.6	0.01

Statistical significance: *p* for M_0_ < 0.001, adjusted R^2^ = 0.0, RMSE = 1.1; *p* for M_1_ < 0.001, adjusted R^2^ = 0.6, RMSE = 0.6; *p* for M_2_ < 0.001, adjusted R^2^ = 0.7, RMSE = 0.5; *p* for M_3_ < 0.001, adjusted R^2^ = 0.7, RMSE = 0.5. Note: The following covariates were considered but not included: Δ TyG, Δ BMI (kg/m^2^), Δ Waist (cm), Δ Weight (kg), Δ Conicity index, Δ VAI, Δ SII (×10^3^), Δ LAP, Δ EAT, Δ HbA1c (%), Δ HDLc (mg/dL), Δ LVEF (%), Δ s′ septal, Δ s′ lateral, Δ IVS (cm), Δ TG (mg/dL), Δ Uric acid (mg/dL), and Δ CT (mg/dL).

## Data Availability

Supporting information is currently available upon request, as long as it keeps the patient’s personal data confidential.

## Abbreviations

The following abbreviations are used in this manuscript:

HF	Heart failure
T2DM	Type 2 diabetes mellitus
GLS	Global longitudinal strain
CVD	Cardiovascular disease
ACC	American College of Cardiology
AHA	American Heart Association
PHF	Pre-heart failure
LV	Left ventricular
TDI	Tissue Doppler Imaging
2D-STE	Two-dimensional speckle-tracking echocardiography
LVEF	Left ventricular ejection fraction
eGFR	Estimated glomerular filtration rate
BMI	Body mass index
HbA1c	Glycated hemoglobin A1c
UACr	Urinary albumin/creatinine ratio
TG	Triglycerides
HDLc	High-density lipoprotein cholesterol
LDLc	Low-density lipoprotein cholesterol
TC	Total cholesterol
LAP	Lipid accumulation product
CRR	Cardiac risk ratio
AC	Atherogenic coefficient
TyG	Product of triglycerides and glucose
VAI	Visceral adiposity index
ECG	Electrocardiogram
IVS	Interventricular septum diameter
PWT	Posterior wall thickness
LVEDD	Left ventricular end-diastolic diameter
LA	Left atrial diameter
LAv	Left atrial volume
EAT	Epicardial fat tissue
LVEF	Left ventricular ejection fraction
S′	Mitral annulus’s systolic velocity
ASE	The American Society of Echocardiography
EACVI	European Association of Cardiovascular Imaging
TAPSE	Tricuspid annular plane systolic excursion
PAAT	Pulmonary artery acceleration time
PV	Peak velocity
GDPR	General Data Protection Regulation
RMSE	Root mean square error
RV	Right ventricle
AOx	Aortic cross
PV PA	Pulmonary artery peak velocity
LVEDV	Left ventricle end-diastolic volume
DTE	E deceleration time
PV Ao	Peak velocity aorta
MV	Mitral valve
AC	Atherogenic coefficient
NLR	Neutrophil-to-lymphocyte ratio
PLR	Platelet-to-lymphocyte ratio
SII	Systemic immune-inflammation index
FPG	Fasting plasma glucose
Δ	The difference between baseline and one-year follow-up
GLP-1RA	Glucagon-like peptide-1 receptor agonist
GLP1-R	GLP-1 receptors
HFpEF	Heart Failure with Preserved Ejection Fraction
ASCVD	Atherosclerotic cardiovascular disease
